# Comparisons between two adapted versions of the Rey Auditory Verbal Learning Test in Brazilian adults: Effects of age and education

**DOI:** 10.1111/jnp.70020

**Published:** 2025-11-05

**Authors:** Luiza Cury Muller, Maria Joana Mäder‐Joaquim, Luciano de Paola, Carlos Eduardo Soares Silvado

**Affiliations:** ^1^ Federal University of Paraná Curitiba PR Brazil

**Keywords:** adult, age factors, Brazil, episodic memory, neuropsychological tests

## Abstract

Reassessments with the Rey Auditory Verbal Learning Test (RAVLT) may generate learning effects, compromising the validity of the results. In Brazil, there are still no comparative studies between adapted versions of the test in healthy individuals. This study compared scores obtained on versions A and B of the RAVLT‐A, routinely used in the neuropsychological assessment of patients with epilepsy and investigated the effects of age, education and version used. A prospective study with 188 cognitively healthy adults was randomly assigned to two groups (version A or B). Comparative analyses between groups and multivariate linear regression models were conducted to examine the impact of age, education and version on RAVLT‐A scores. No significant differences were observed between versions A and B of the RAVLT‐A (*p* > .05). Regression indicated a significant influence of age and, especially, education on performance on the test variables. The version used had no statistically relevant impact on the scores. This is the first Brazilian study to examine two adapted versions of the RAVLT‐A in healthy adults. The findings demonstrate comparability between versions A and B, supporting their alternate use in reassessments to reduce practice effects. Age and, especially, education significantly influenced performance, emphasizing the need for normative data stratified by both variables, since those commonly used in Brazil are stratified only by age.

## INTRODUCTION

In neuropsychological practice, it is common to reapply tests during clinical and intervention reassessments (Heilbronner et al., 2010). However, repeated administration can result in learning effects (Holm et al., 2022; Kremen et al., 2022), defined by improved test performance resulting from repeated exposure to the material or procedure, rather than an improvement in the cognitive function being assessed (Calamia et al., 2012; Heilbronner et al., 2010; Salthouse, 2012). The use of alternative versions of classic memory tests is a practice widely recommended in the literature to minimize these effects (Mitrushina et al., 2005; Nguyen et al., 2024; Rasmussen et al., 2001; Zucchella et al., 2018). However, there are still few Brazilian studies with adapted versions of classic tests.

In this sense, one of the tests susceptible to this effect is the Rey Auditory Verbal Learning Test (RAVLT) (Rey, 1958), which involves the serial presentation of a list of words to assess auditory‐verbal episodic memory. Repeating the same list can compromise the accuracy of the assessment, making it difficult to accurately identify the cognitive profile (Gavett et al., 2016; Goldberg et al., 2015; Sanderson‐Cimino et al., 2023). In addition, this effect can distort cognitive estimates in longitudinal studies (Elman et al., 2018; Freedman & Hu, 2024; Salthouse, 2019).

The international literature includes both adaptations of the RAVLT for different linguistic and cultural contexts (Gottlieb et al., 2022, 2024; Malyutina et al., 2024; Smirni et al., 2018) as well as the development of parallel forms designed to reduce reapplication effects (Fastenau et al., 2002; Lee Meeuw Kjoe et al., 2022). In Brazil, although lists adapted for Portuguese have been published (De Paula et al., 2018; Malloy‐Diniz et al., 2000, 2007; Noffs et al., 2002), to date, there are no comparative studies involving two different versions of the RAVLT test applied to a sample of healthy individuals. This absence compromises the validity of clinical reassessments in Brazil and justifies the need for studies that verify the comparability between adapted versions.

The main objective of this study was to compare performance on versions A and B of the Rey Adapted Auditory Verbal Learning Test (RAVLT‐A) in a sample of healthy adults from the Curitiba region (PR) and to investigate whether age, education and test version significantly influence performance on the RAVLT‐A, using linear regression models. In addition, we sought to provide preliminary data, creating a comparative basis for clinical interpretation and encouraging the use of these alternative forms in different care settings.

The versions of the RAVLT‐A analysed are part of the neuropsychological protocol used for over 30 years in the Epilepsy Surgery Program at the Federal University of Parana Clinical Hospital (Jamus et al., 2023; Mader, 2004; Mäder, 2001; Mader et al., 2001; Muller et al., 2021; Ono et al., 2019, 2021).

## MATERIALS AND METHODS

### Transparency and openness

Our study complies with the transparency and openness promotion guidelines. We report how we determined our sample size, all exclusions, manipulations and measures. Raw and processed data, as well as the syntax used in the analyses, are available upon reasonable request from the corresponding author, in accordance with ethical procedures for the reuse of sensitive data. The study's design and analyses were not preregistered.

### Individuals

A prospective descriptive study was conducted to characterize a sample of cognitively healthy adults from Curitiba (PR) and its metropolitan area. Participants were recruited through a non‐probabilistic sample, based on social media posts, institutional contacts and spontaneous referrals between 2022 and 2024. The sample consisted of 188 Brazilian, native Portuguese‐speaking, individuals aged between 18 and 59 years, literate and without visual, cognitive or motor difficulties that could interfere with the performance of neuropsychological tests. This age range was selected because the adapted RAVLT‐A versions are routinely applied in the neuropsychological protocol of the Epilepsy Surgery Program, where adults in this age group represent the most frequent demographic profile assessed. The sample size was determined by prior calculation, estimating a minimum requirement of 182 participants to detect a difference of .5 points between the versions of the RAVLT‐A, with 80% power and 5% alpha.

Participants diagnosed with active neuropsychiatric diseases or undergoing uncontrolled treatment, or self‐reported use of illegal drugs, scores above the cut‐off point (≥12 for anxiety and/or depression) on the Hospital Anxiety and Depression Scale (HADS), history of admission to an intensive care unit (ICU) due to COVID‐19 or recurrent school failure (two or more times) were excluded. Individuals using antiepileptic medication were also excluded. These criteria were intended to ensure a sample composed of cognitively healthy and functionally independent individuals.

### Sample division

Participants were randomly divided into two groups: Group 1 (RAVLT‐A version A) and Group 2 (RAVLT‐A version B). Each group was stratified into subgroups according to age (<40 years and ≥40 years) and educational level. Educational level was stratified into three ranges: up to 9 years (elementary school), 10–12 years (high school) and more than 12 years (higher education). This classification was established based on the Brazilian educational structure, which consists of elementary, high school and higher education levels, respectively.

### Evaluation procedure

All participants were informed about the study objectives, signed an informed consent form and completed an initial assessment in person, which included the collection of sociodemographic and health data, with an emphasis on screening for signs of anxiety and depression using the Hospital Anxiety and Depression Scale (HADS). The use of this instrument was justified by its validation and standardization for the Brazilian population as well as its widespread application as a brief screening tool in community and non‐clinical samples. Next, a standardized neuropsychological protocol was applied, consisting of instruments for assessing executive functioning, visuospatial episodic memory and auditory‐verbal memory, the latter represented by versions A or B of the Rey Adapted Auditory‐Verbal Learning Test (RAVLT‐A).

### Instruments

The Rey Adapted Auditory‐Verbal Learning Test (RAVLT‐A), versions A and B, assesses auditory‐verbal episodic memory through the oral presentation of a list of 15 words (list A), read aloud by the examiner in five consecutive attempts (A1–A5). After each reading, the participant is asked to verbally recall as many words as possible. Next, an interference list (list B) is presented (B1), and the participant is then asked to recall list A (A6). After a 20‐min interval, the delayed recall of list A (A7) is applied. Attempts A1–A5 comprise the learning curve, whereas A7 assesses delayed retention.

Versions A and B used in this study are part of the neuropsychological protocol of the Epilepsy Surgery Program at the Federal University of Parana Clinical Hospital (Mader, 2004; Mader et al., 2001) and are routinely applied in the pre‐ and post‐surgical context, respectively. They are adaptations of the original Rey test (Rey, 1958), differing only in the content of the word lists.

### Statistical analysis

Results of quantitative variables were described in terms of mean and standard deviation. Categorical variables were described by frequency and percentage. Fisher's exact test or the Chi‐square test was used to compare Groups 1 and 2, age groups (<40 or ≥40 years) and educational classifications (≤9, 10–12 or >12 years). Quantitative variables were analysed using Student's *t*‐test for independent samples or the nonparametric Mann–Whitney test (2 groups). Effect sizes (Cohen's *d* for *t*‐tests and Cramer's *V* for chi‐squared tests) with 95% confidence intervals were calculated. Pearson's linear correlation coefficient or Spearman's correlation coefficient was estimated to assess the correlation between two quantitative variables.

Multivariate linear regression models were adjusted to assess the joint effect of age group, educational level and RAVLT‐A versions on neuropsychological test results. Initially, interaction terms between the three independent variables were included to explore possible interactive effects. Considering that no significant interactions were found, the final models were adjusted with only the main effects. The normality of the continuous variables was assessed using the Kolmogorov–Smirnov test. Values of *p* < .05 indicated statistical significance (IBM SPSS Statistics v.29.0).

## RESULTS

The sociodemographic characteristics of the sample, including gender, age, education level, manual dominance and the distribution of participants by age and education subgroups, are presented in Table 1. Statistical analyses indicated that groups 1 (version A; *n* = 91) and 2 (version B; *n* = 97) were homogeneous in all quantitative variables (age and education) and categorical variables (age group <40 and ≥40 years, combined age and education strata, gender and manual dominance), with *p*‐values > .05.

**TABLE 1:** Summary of demographic data and homogeneity between groups.

Variable	Group 1 (*n* = 91)		Group 2 (*n* = 97)		*p*
Age | mean (SD)	38.0 (10.5)		37.7 (11.5)		.889^1^
Education level | mean (SD)	12.4 (4.3)		12.3 (4.3)		.747^1^
Age (years)	**(*n*)**	**%**	**(*n*)**	**%**	.465^2^
<40	45	49.50%	54	55.70%	
≥40	46	50.50%	43	44.30%	
Subgroups (age, education level)					.948^2^
<40, education level ≤9	11	12.10%	13	13.4%	
<40, education level 9–12	18	19.80%	22	22.70%	
<40, education level >12	16	17.60%	19	19.60%	
≥40, education level ≤9	13	14.30%	14	14.40%	
≥40, education level 9–12	15	16.50%	15	15.50%	
≥40, education level >12	18	19.80%	14	14.40%	
Sex					.561^2^
Male	51	56.00%	50	51.50%	
Female	40	44.00%	47	48.50%	
Education level (years)					.892^2^
≤9	24	26.40%	27	27.80%	
10–12	33	36.30%	37	38.10%	
>12	34	37.40%	33	34.00%	
Handedness					–
Right	84	92.30%	92	94.80%	
Left	7	7.70%	4	4.10%	
Ambidextrous	0	.00%	1	1.00%	

*Note*: ^1^Student's *t*‐test for independent samples or Mann–Whitney non‐parametric test, *p* < .05; ^2^Fisher's exact test or chi‐squared test, *p* < .05. No statistically significant group differences were found across demographic variables.

Table 2 presents descriptive statistics (mean and standard deviation) of the RAVLT‐A variables, stratified by age group (<40 years and ≥40 years) and educational level (≤9 years, 10–12 years and >12 years). Analysis of these data indicated significant effects of age and education on the scores obtained, with better performance among younger participants with higher education levels.

**TABLE 2:** Descriptive statistics of RAVLT‐A (versions A and B) stratified by age and education level.

Education level	<40 years						≥40 years					
	<9		10–12		12+		<9		10–12		12+	
	Mean	SD	Mean	SD	Mean	SD	Mean	SD	Mean	SD	Mean	SD
RAVLT‐A v.A												
A1	4.9	1.1	4.8	1.9	5.6	1.3	3.6	1.4	3.8	1.3	5.2	1
A2	7.5	1.2	7.1	2.5	9.2	1.8	4.7	1.4	6.5	1.6	7.9	1.8
A3	8.2	1.5	8.9	1.9	10.8	2.4	5.8	1.6	8.5	1.2	9.4	2.7
A4	8.5	1.8	10	1.8	11.7	2.1	7.1	1.7	9.2	1.3	10.9	2.1
A5	9.3	1.3	10.7	1.7	12.1	1.9	7.2	1.3	10.1	2.1	11.4	1.8
SUM (A1–A5)	38.5	4.9	41.6	8.7	49.3	7.9	28.6	5.6	38	5.3	44.9	8
B1	4.1	1.9	4.2	1.7	6.2	1.2	3.1	.8	3.8	1.4	4.3	1
A6	7.6	1.4	9.2	2	11	3.2	5.4	1.3	8	1.7	9.4	2.7
A7	7.5	2.3	8.8	2.1	11	2.9	5.7	1.2	8.5	1.7	9.7	2.3
RAVLT‐A v.B												
A1	4.3	1.1	5	1.4	5.1	1.4	3.6	1	4.5	.9	5.2	1.3
A2	6.4	1.8	7.5	1.6	8	1.9	5.9	1.2	6	1.6	7.3	1.4
A3	7.2	1.4	9.1	2.2	9.8	1.8	6.6	1.5	8	1.7	8.8	2.1
A4	8.2	2	10.6	1.6	11.6	3.5	8.1	1.9	8.4	1.8	9.8	1.6
A5	8.7	1.8	11.5	1.3	11.6	2.3	8.4	1.5	8.7	1.9	10.7	2
SUM (A1–A5)	34.7	7	43.7	5.3	46.1	8.3	32.5	5.1	35.6	6.7	41.8	7.1
B1	3.9	1.5	4.6	1.2	5.1	1.6	2.4	1.2	3.7	.8	4.3	1.6
A6	7.2	2.2	9.4	2.1	10.2	2.7	6.7	1.9	6.7	1.6	8.2	2.1
A7	6.9	1.4	9.5	2.1	10.5	2.2	7.1	1.4	7.3	1.3	8.9	2.1

Abbreviations: RAVLT‐A v.A, Rey Auditory Verbal Learning Test‐Adapted (version A); RAVLT‐A v.B, Rey Auditory Verbal Learning Test‐Adapted (version B); SUM (A1–A5), sum of the total scores of the five trials (A1–A5).

Direct comparison between the scores obtained in versions A and B of the RAVLT‐A did not reveal statistically significant differences in any of the variables analysed (*p* > .05), with no meaningful effect sizes. Table 3 summarizes the descriptive statistics by version and the *p*‐values for the comparison tests.

**TABLE 3:** Comparison between RAVLT‐A results (version A and B) considering all individuals.

RAVLT‐A	Version A (*n* = 91)		Version B (*n* = 97)		*p*
	Mean	SD	Mean	SD	
A1	4.7	1.5	4.7	1.3	.873
A2	7.2	2.2	6.9	1.7	.336
A3	8.7	2.4	8.4	2.1	.299
A4	9.8	2.3	9.6	2.5	.739
A5	10.3	2.3	10.2	2.3	.643
SUM (A1–A5)	40.8	9.4	39.8	8.2	.458
B1	4.3	1.6	4.1	1.5	.305
A6	8.6	2.8	8.3	2.5	.366
A7	8.7	2.7	8.6	2.3	.751

*Note*: Student's *t*‐test for independent samples, *p* < .05. No significant differences were found between versions A and B across all RAVLT‐A trials (all |*t*|(186) ≤ 1.05, all *p* ≥ .295). Effect sizes were minimal (Cohen's *d* range = .00–.15, 95% CIs all including zero).

Abbreviations: RAVLT‐A, Rey Auditory Verbal Learning Test‐Adapted; SD, standard deviation; SUM (A1–A5), Sum of the total scores of the five trials (A1–A5).

Linear regression models adjusted for RAVLT‐A variables revealed consistent associations between age, education and performance on SUM (A1–A5, learning curve) and A7 (delayed recall). The analyses showed that younger participants (<40 years) and those with higher education (>12 years) obtained significantly higher scores on both variables. This effect was more pronounced in SUM (A1–A5), with a mean difference of 5.29 points between participants under 40 years and those aged 40 years or older (*p* < .001). Regarding education, participants with 10–12 years of schooling had mean scores 6.33 points higher than those in the group with ≤9 years (*p* < .001), while those with more than 12 years of schooling had, on average, 11.98 points more than the reference group (*p* < .001).

No significant associations were found between the versions, indicating that the differences between forms A and B of the RAVLT‐A do not significantly influence the results adjusted for age and schooling, both in A7 (*p* = .701) and in SUM (A1–A5) (*p* = .323). The estimated age and education coefficients, their 95% confidence intervals, and *p*‐values are presented in Table S1.

## DISCUSSION

Our first set of descriptive results indicates a significant influence of age and education on RAVLT‐A scores. In general, regardless of age group, participants with higher educational levels (>12 years) had higher scores. These findings are consistent with the literature, which highlights education as a factor associated with higher levels of performance on auditory‐verbal episodic memory tests (Dassanayake et al., 2020; Espenes et al., 2023; Messinis et al., 2016) as well as in various cognitive domains throughout the course of development (Harrsen et al., 2021; Lövdén et al., 2020; Rehnberg et al., 2024), which reinforces the trend observed in our analysis.

Our second group of results indicates no statistically significant differences (*p*‐values > .05) in the means of all RAVLT‐A variables between versions A and B, with similar mean scores between them. This performance pattern provides initial evidence of comparability between the adapted versions of the RAVLT‐A when applied to a Brazilian sample of healthy adults. These results are in line with the international literature, which describes the development of alternative versions of the RAVLT as a strategy to minimize learning effects resulting from repetition of the instrument (Beglinger et al., 2005; Bezdicek et al., 2014; Boenniger et al., 2021; Fastenau et al., 2001, 2002; Lee Meeuw Kjoe et al., 2022; Malyutina et al., 2024; Paštrnák et al., 2018).

The results obtained contribute to the construction of a preliminary database useful for clinical interpretation and comparison of performance scores, especially in the context of pre‐ and post‐surgical neuropsychological assessment of epilepsy, for which these versions were originally developed (Mader, 2004; Mader et al., 2001). Furthermore, the demonstration of consistency between versions A and B of the RAVLT‐A reinforces its applicability in different care and research contexts, especially in situations that require serial reassessments and control of learning effects (Campos‐Magdaleno et al., 2017; Duff et al., 2018; Duff & Hammers, 2022; Gavett et al., 2016). Our findings provide clinicians with empirical support to administer version A at the beginning of an evaluation process and version B in follow‐up assessments, ensuring consistency and comparability across repeated testing.

Linear regression analyses indicated that age has a greater impact on the learning curve (represented by SUM A1–A5) than on delayed recall (A7), which can be attributed to the different cognitive demands involved in each stage of the task. While delayed recall depends predominantly on mechanisms of retention and retrieval of previously consolidated information, with less involvement of cognitive strategies (Bezdicek et al., 2014; Karakaş et al., 2022; Messinis et al., 2016; Saury & Emanuelson, 2017; Witt et al., 2019), the learning curve recruits multiple skills during sequential encoding, such as sustained attention, semantic organization, categorisation, response monitoring and processing speed—functions mediated by executive components that are particularly sensitive to the ageing process (Almkvist et al., 2025; Constantinidou et al., 2014; Dörr et al., 2023; Espenes et al., 2023; Messinis et al., 2016; Putcha et al., 2019).

This functional complexity may explain the greater sensitivity of the learning curve to the effects of age, reflecting its combined dependence on the integrity of memory‐related anatomical structures and strategic resources mediated by executive functions (Constantinidou et al., 2014; Espenes et al., 2023; Messinis et al., 2016; Saury & Emanuelson, 2017; Witt et al., 2019). In contrast, delayed recall tends to remain relatively preserved in typical aging and acts as a marker less susceptible to cognitive variations in healthy adults (Bezdicek et al., 2014; Karakaş et al., 2022; Messinis et al., 2016; Witt et al., 2019).

Previous studies highlight age as one of the main predictors of performance in adapted versions of the RAVLT (Bezdicek et al., 2014; Dassanayake et al., 2020; De Paula et al., 2018; Espenes et al., 2023; Messinis et al., 2016; Speer et al., 2014), whereas in the present study, education level had a more significant impact on performance in the sample group studied. These results can be explained by Brazilian sociocultural issues, in which the level of formal education achieved by an individual in Brazil does not always reflect the same educational skills observed in other countries (Gonçalves et al., 2023, 2025; Seblova et al., 2020).

Basic and higher education in the Brazilian context is highly heterogeneous, with marked regional and socioeconomic disparities that affect the quality of teaching and the development of cognitive skills. Although many individuals complete several years of formal education, functional literacy levels often remain below expectations for their reported schooling, indicating that academic attainment does not uniformly translate into equivalent cognitive outcomes (Gonçalves et al., 2023). As a result, individuals with the same number of years of schooling may present different levels of proficiency due to these structural inequalities (Kochhann et al., 2009, 2010; Lima et al., 2023; Schwartzman, 2004). Thus, we highlight the importance of considering the factor of schooling in the neuropsychological analyses of this test, since the normative data commonly used in Brazil present parameters stratified only by age (De Paula et al., 2018; Malloy‐Diniz et al., 2000, 2007).

This study has some limitations that should be considered when interpreting the results. The distribution of participants across different age groups and educational levels may have limited the statistical power to detect the impact of age, especially when compared to studies with larger samples. These factors reinforce the need for future studies with larger, more representative samples stratified by age and education level from different regions of Brazil, in order to allow generalization to representative data at the national level. Moreover, as participants in this study were aged between 18 and 59 years, the findings from the comparison between the adapted RAVLT‐A versions cannot be extrapolated to individuals over 60 years of age.

This is the first Brazilian study to apply two alternative versions of the RAVLT‐A in a sample of healthy individuals. The results indicate that both versions of the test (A and B) provide consistent results and can therefore be used to minimize learning effects in neuropsychological reassessments. Our results highlight the significant influence of age and, especially, education on RAVLT‐A performance, emphasizing the impact of Brazilian sociocultural factors.

## AUTHOR CONTRIBUTIONS

**Luiza Cury Muller:** Conceptualization; methodology; validation; writing – review and editing; writing – original draft; data curation; formal analysis. **Maria Joana Mäder‐Joaquim:** Supervision; writing – review and editing; visualization; validation; methodology; project administration. **Luciano de Paola:** Conceptualization; methodology; writing – review and editing. **Carlos Eduardo Soares Silvado:** Conceptualization; methodology; validation; writing – review and editing; supervision; project administration.

## CONFLICT OF INTEREST STATEMENT

The authors declare no competing financial interests or personal relationships that could have appeared to influence the work reported in this paper.

### ETHICS STATEMENT

This research was approved by the Ethics Committee for Research on Human Beings of the Federal university of Parana Clinical Hospital (certificate number: 37913020.4.0000.0096; protocol: 4.442.082). Written informed consent was obtained from all participants. All procedures followed the Declaration of Helsinki and institutional guidelines.

REFERENCES

Almkvist, O.
, 
Rennie, A.
, 
Westman, E.
, 
Wallert, J.
, & 
Ekman, U.
 (2025). Methods for assessment of Rey Auditory Verbal Learning Test performance in memory clinic patients and healthy adults—At the cross‐roads of learning theory and clinical utility. The Clinical Neuropsychologist, 39(2), 424–438. 10.1080/13854046.2024.2384616
39135427


Beglinger, L. J.
, 
Gaydos, B.
, 
Tangphao‐Daniels, O.
, 
Duff, K.
, 
Kareken, D. A.
, 
Crawford, J.
, 
Fastenau, P. S.
, & 
Siemers, E. R.
 (2005). Practice effects and the use of alternate forms in serial neuropsychological testing. Archives of Clinical Neuropsychology, 20(4), 517–529. 10.1016/j.acn.2004.12.003
15896564


Bezdicek, O.
, 
Stepankova, H.
, 
Moták, L.
, 
Axelrod, B. N.
, 
Woodard, J. L.
, 
Preiss, M.
, 
Nikolai, T.
, 
Růžička, E.
, & 
Poreh, A.
 (2014). Czech version of Rey Auditory Verbal Learning Test: Normative data. Aging, Neuropsychology, and Cognition, 21(6), 693–721. 10.1080/13825585.2013.865699
24344673

Boenniger, M. M.
, 
Staerk, C.
, 
Coors, A.
, 
Huijbers, W.
, 
Ettinger, U.
, & 
Breteler, M. M. B.
 (2021). Ten German versions of Rey's auditory verbal learning test: Age and sex effects in 4,000 adults of the Rhineland study. Journal of Clinical and Experimental Neuropsychology, 43(6), 637–653. 10.1080/13803395.2021.1984398
34636711


Calamia, M.
, 
Markon, K.
, & 
Tranel, D.
 (2012). Scoring higher the second time around: Meta‐analyses of practice effects in neuropsychological assessment. The Clinical Neuropsychologist, 26(4), 543–570. 10.1080/13854046.2012.680913
22540222


Campos‐Magdaleno, M.
, 
Facal, D.
, 
Lojo‐Seoane, C.
, 
Pereiro, A. X.
, & 
Juncos‐Rabadán, O.
 (2017). Longitudinal assessment of verbal learning and memory in amnestic mild cognitive impairment: Practice effects and meaningful changes. Frontiers in Psychology, 8, 1231. 10.3389/fpsyg.2017.01231
28775700
PMC5518168

Constantinidou, F.
, 
Zaganas, I.
, 
Papastefanakis, E.
, 
Kasselimis, D.
, 
Nidos, A.
, & 
Simos, P. G.
 (2014). Age‐related decline in verbal learning is moderated by demographic factors, working memory capacity, and presence of amnestic mild cognitive impairment. Journal of the International Neuropsychological Society, 20(8), 822–835. 10.1017/S1355617714000678
25156204


Dassanayake, T. L.
, 
Hewawasam, C.
, 
Baminiwatta, A.
, 
Samarasekara, N.
, & 
Ariyasinghe, D. I.
 (2020). Sex‐, age‐ and education‐adjusted norms for the WHO/UCLA version of the Rey Auditory Verbal Learning Test for Sinhala‐speaking Sri Lankan adults. The Clinical Neuropsychologist, 34(sup1), 127–142. 10.1080/13854046.2020.1829069
33025851


De Paula, J. J.
, 
Fernandes, C. S.
, & 
Malloy‐Diniz, L. F.
 (2018). 
Teste de aprendizagem auditivo‐verbal de Rey (RAVLT). Vetor editora psico‐Pedagógica
.

Dörr, F.
, 
Schäfer, S.
, 
Öhman, F.
, 
Linz, N.
, 
Bodin, T. H.
, 
Skoog, J.
, 
Zettergren, A.
, 
Kern, S.
, 
Skoog, I.
, & 
Tröger, J.
 (2023). Dissociating memory and executive function impairment through temporal features in a word list verbal learning task. Neuropsychologia, 189, 108679. 10.1016/j.neuropsychologia.2023.108679
37683887


Duff, K.
, 
Anderson, J. S.
, 
Mallik, A. K.
, 
Suhrie, K. R.
, 
Atkinson, T. J.
, 
Dalley, B. C. A.
, 
Morimoto, S. S.
, & 
Hoffman, J. M.
 (2018). Short‐term repeat cognitive testing and its relationship to hippocampal volumes in older adults. Journal of Clinical Neuroscience, 57, 121–125. 10.1016/j.jocn.2018.08.015
30143414
PMC6191314

Duff, K.
, & 
Hammers, D. B.
 (2022). Practice effects in mild cognitive impairment: A validation of Calamia et al. (2012). The Clinical Neuropsychologist, 36(3), 571–583. 10.1080/13854046.2020.1781933
32594886
PMC7765738

Elman, J. A.
, 
Jak, A. J.
, 
Panizzon, M. S.
, 
Tu, X. M.
, 
Chen, T.
, 
Reynolds, C. A.
, 
Gustavson, D. E.
, 
Franz, C. E.
, 
Hatton, S. N.
, 
Jacobson, K. C.
, 
Toomey, R.
, 
McKenzie, R.
, 
Xian, H.
, 
Lyons, M. J.
, & 
Kremen, W. S.
 (2018). Underdiagnosis of mild cognitive impairment: A consequence of ignoring practice effects. Alzheimer's & Dementia: Diagnosis, Assessment & Disease Monitoring, 10, 372–381. 10.1016/j.dadm.2018.04.003
PMC603970830003138

Espenes, J.
, 
Eliassen, I. V.
, 
Öhman, F.
, 
Hessen, E.
, 
Waterloo, K.
, 
Eckerström, M.
, 
Lorentzen, I. M.
, 
Bergland, C.
, 
Niska, M. H.
, 
Timón‐Reina, S.
, 
Wallin, A.
, 
Fladby, T.
, & 
Kirsebom, B. E.
 (2023). Regression‐based normative data for the Rey Auditory Verbal Learning Test in Norwegian and Swedish adults aged 49–79 and comparison with published norms. The Clinical Neuropsychologist, 37(6), 1276–1301. 10.1080/13854046.2022.2106890
35968846


Fastenau, P. S.
, 
Hankins, W. T.
, & 
McGinnis, C. M.
 (2001). Content validity of five alternate forms for six established neuropsychological tests (abstract). Archives of Clinical Neuropsychology, 16(8), 824.

Fastenau, P. S.
, 
Hankins, W. T.
, 
McGinnis, C. M.
, 
Moy, T.
, & 
Richard, M.
 (2002). Effects of alternate forms on retest effects in clinical testing. Journal of the International Neuropsychological Society, 8, 151–161.

Freedman, V. A.
, & 
Hu, M.
 (2024). Addressing practice effects in population‐based studies of trends in late‐life dementia and cognitive impairment. The Journals of Gerontology, Series A: Biological Sciences and Medical Sciences, 79(Suppl. 1), S7–S10. 10.1093/gerona/glae198
39132707
PMC11542059

Gavett, B. E.
, 
Gurnani, A. S.
, 
Saurman, J. L.
, 
Chapman, K. R.
, 
Steinberg, E. G.
, 
Martin, B.
, 
Chaisson, C. E.
, 
Mez, J.
, 
Tripodis, Y.
, & 
Stern, R. A.
 (2016). Practice effects on story memory and list learning tests in the neuropsychological assessment of older adults. PLoS One, 11(10), e0164492. 10.1371/journal.pone.0164492
27711147
PMC5053775

Goldberg, T. E.
, 
Harvey, P. D.
, 
Wesnes, K. A.
, 
Snyder, P. J.
, & 
Schneider, L. S.
 (2015). Practice effects due to serial cognitive assessment: Implications for preclinical Alzheimer's disease randomized controlled trials. Alzheimer's & Dementia, 1(1), 103–111. 10.1016/j.dadm.2014.11.003
PMC487690227239497

Gonçalves, N. G.
, 
Avila, J. C.
, 
Bertola, L.
, 
Obregón, A. M.
, 
Ferri, C. P.
, 
Wong, R.
, & 
Suemoto, C. K.
 (2023). Education and cognitive function among older adults in Brazil and Mexico. Alzheimer's & Dementia, 15, 1–9. 10.1002/dad2.12470
PMC1052345237771429

Gonçalves, N. G.
, 
Paradela, R. S.
, 
Avila, J. C.
, 
Bertola, L.
, 
Ferri, C. P.
, 
Wong, R.
, & 
Suemoto, C. K.
 (2025). Rural‐urban disparities in cognitive performance in Brazil and Mexico. Aging and Mental Health, 29, 1814–1820. 10.1080/13607863.2025.2502780
40396337


Gottlieb, A.
, 
Doniger, G. M.
, 
Kimel‐Naor, S.
, 
Ben‐Gal, O.
, 
Cohen, M.
, 
Iny, H.
, 
Beeri, M. S.
, & 
Plotnik, M.
 (2022). Development and validation of virtual reality‐based Rey Auditory Verbal Learning Test. Frontiers in Aging Neuroscience, 14, 980093. 10.3389/fnagi.2022.980093
36185470
PMC9519387

Gottlieb, A.
, 
Kimel‐Naor, S.
, 
Zeilig, G.
, 
Schnaider Beeri, M.
, & 
Plotnik, M.
 (2024). Assessment of verbal memory in Parkinson's disease utilizing a virtual reality‐based Rey Auditory Verbal Learning Test. Scientific Reports, 14(1), 21792. 10.1038/s41598-024-71618-6
39294213
PMC11410795

Harrsen, K.
, 
Christensen, K.
, 
Lund, R.
, & 
Mortensen, E. L.
 (2021). Educational attainment and trajectories of cognitive decline during four decades‐the Glostrup 1914 cohort. PLoS One, 16(8), e0255449. 10.1371/journal.pone.0255449
34339478
PMC8328320

Heilbronner, R. L.
, 
Sweet, J. J.
, 
Attix, D. K.
, 
Krull, K. R.
, 
Henry, G. K.
, & 
Hart, R. P.
 (2010). Official position of the American Academy of clinical neuropsychology on serial neuropsychological assessments: The utility and challenges of repeat test administrations in clinical and forensic contexts. The Clinical Neuropsychologist, 24(8), 1267–1278. 10.1080/13854046.2010.526785
21108148


Holm, S. P.
, 
Wolfer, A. M.
, 
Pointeau, G. H. S.
, 
Lipsmeier, F.
, & 
Lindemann, M.
 (2022). Practice effects in performance outcome measures in patients living with neurologic disorders–A systematic review. Heliyon, 8(8), e10259. 10.1016/j.heliyon.2022.e10259
36082322
PMC9445299

Jamus, D. R.
, 
Mäder‐Joaquim, M. J.
, 
de Paula Souza, L.
, 
Paola, L.
, 
Claro‐Höpker, C. D.
, 
Terra, V. C.
, & 
Soares Silvado, C. E.
 (2023). Rey‐Osterrieth complex figure test: Comparison of traditional and qualitative scoring systems after unilateral temporal lobectomy. The Clinical Neuropsychologist, 37(2), 416–431. 10.1080/13854046.2022.2047790
35264077


Karakaş, S.
, 
Erdoğan Bakar, E.
, 
Doğutepe, E.
, 
Can, H.
, & 
Kaskatı, T.
 (2022). Differentiation of memory processing stages and effect of demographic variables with alternative scoring approaches to the Rey Auditory Verbal Learning Test. Journal of Clinical and Experimental Neuropsychology, 44(2), 109–133. 10.1080/13803395.2022.2080186
35670663


Kochhann, R.
, 
Cerveira, M. O.
, 
Godinho, C.
, 
Camozzato, A.
, & 
Chaves, M. L. F.
 (2009). Evaluation of mini‐mental state examination scores according to different age and education strata, and sex, in a large Brazilian healthy sample. Dementia and Neuropsychologia, 3(2), 88–93. 10.1590/S1980-57642009DN30200004
29213617
PMC5619224

Kochhann, R.
, 
Varela, J. S.
, 
Lisboa, C. S. M.
, & 
Chaves, M. L. F.
 (2010). The mini mental state examination: Review of cutoff points adjusted for schooling in a large southern Brazilian sample. Dementia and Neuropsychologia, 4(1), 35–41. 10.1590/S1980-57642010DN40100006
29213658
PMC5619528

Kremen, W. S.
, 
Nation, D. A.
, & 
Nyberg, L.
 (2022). Editorial: The importance of cognitive practice effects in aging neuroscience. Frontiers in Aging Neuroscience, 14, 1079021. 10.3389/fnagi.2022.1079021
36466612
PMC9716314

Lee Meeuw Kjoe, P. R.
, 
Vermeulen, I. E.
, 
Agelink van Rentergem, J. A.
, 
van der Wall, E.
, & 
Schagen, S.
 (2022). Standardized item selection for alternate computerized versions of Rey Auditory Verbal Learning Test(−based) word lists. Journal of Clinical and Experimental Neuropsychology, 44(9), 681–701. 10.1080/13803395.2023.2166904
36660813


Lima, D. P.
, 
Bonfadini, J. d. C.
, 
Carneiro, A. H. S.
, 
de Almeida, S. B.
, 
Viana, A. B.
, 
Nogueira E Silva, A. C.
, 
de Sa Roriz, J.
, & 
Braga, P.
 (2023). Educational disparities in Brazil may interfere with the cognitive performance of Parkinson's disease patients. Dementia and Neuropsychologia, 17, e20220084. 10.1590/1980-5764-DN-2022-0084
38028380
PMC10666553

Lövdén, M.
, 
Fratiglioni, L.
, 
Glymour, M. M.
, 
Lindenberger, U.
, & 
Tucker‐Drob, E. M.
 (2020). Education and cognitive functioning across the life span. Psychological Science in the Public Interest, 21(1), 6–41. 10.1177/1529100620920576
32772803
PMC7425377

Mäder, M. J.
 (2001). Avaliação neuropsicológica nas epilepsias: Importância para o conhecimento do cérebro. Psicologia: Ciência e Profissão, 21(1), 54–67. 10.1590/S1414-98932001000100007


Mader, M. J.
 (2004). O teste de Wada como indicativo do prognóstico de disfunção de memória após lobectomia temporal anterior em pacientes portadores de epilepsia de lobo temporal unilateral (Tese de doutorado, Universidade de São Paulo). https://pesquisa.bvsalud.org/portal/resource/pt/lil‐397861


Mader, M. J.
, 
Damasceno, B.
, 
Frank, J.
, & 
Portuguez, M.
 (2001). Critérios mínimos para procedimentos de avaliação neuropsicológica pré e pós‐cirúrgica. Journal of Epilepsy and Clinical Neurophysiology, 20017, 104–105.

Malloy‐Diniz, L. F.
, 
Lasmar, V. A.
, 
Gazinelli, L. d. S.
, 
Fuentes, D.
, & 
Salgado, J. V.
 (2007). The Rey auditory‐verbal learning test: Applicability for the Brazilian elderly population. Brazilian Journal of Psychiatry, 29(4), 324–329. 10.1590/s1516-44462006005000053
17713697


Malloy‐Diniz, L. F. M.
, 
Cruz, M. F.
, 
Torres, V. M.
, & 
Cosenza, R. M.
 (2000). O teste de Aprendizagem Auditivo‐Verbal de Rey: Normas para uma população brasileira. Revista Brasileira de Neurologia, 36, 79–83.

Malyutina, S.
, 
Zubareva, N.
, 
Buivolova, O.
, 
Zontov, Y.
, 
Shestakova, E.
, 
Chernova, M.
, 
Bedo, A.
, 
Andriushchenko, A.
, 
Savilov, V.
, 
Kurmysheva, E.
, 
Kibardina, A.
, 
Kotova, N.
, 
Sobko, A.
, 
Akbarova, Z.
, & 
Dragoy, O.
 (2024). The Rey Auditory Verbal Learning Test: Adaptation into Russian and a new digital “RAVLT World”. The Clinical Neuropsychologist, 39, 2106–2128. 10.1080/13854046.2024.2446028
39741380


Messinis, L.
, 
Nasios, G.
, 
Mougias, A.
, 
Politis, A.
, 
Zampakis, P.
, 
Tsiamaki, E.
, 
Malefaki, S.
, 
Gourzis, P.
, & 
Papathanasopoulos, P.
 (2016). Age and education‐adjusted normative data and discriminative validity for Rey's auditory verbal learning test in the elderly Greek population. Journal of Clinical and Experimental Neuropsychology, 38(1), 23–39. 10.1080/13803395.2015.1085496
26588427


Mitrushina, M.
, 
Boone, K. B.
, 
Razani, J.
, & 
Della Pompa, M.
 (2005). Handbook of normative data for neuropsychological assessment (2nd ed.). Oxford University Press.

Muller, L. C.
, 
Mäder‐Joaquim, M. J.
, 
Terra, V. C.
, 
de Paola, L.
, 
Hopker, C. D. C.
, 
de Paula Souza, L.
, & 
Soares Silvado, C. E.
 (2021). Nonverbal fluency assessed by the five‐point test in epilepsy patients with unilateral mesial temporal sclerosis‐A Brazilian study. The Clinical Neuropsychologist, 35(sup1), S21–S31. 10.1080/13854046.2021.1887357
33622173


Nguyen, C. M.
, 
Rampa, S.
, 
Staios, M.
, 
Nielsen, T. R.
, 
Zapparoli, B.
, 
Zhou, X. E.
, 
Mbakile‐Mahlanza, L.
, 
Colon, J.
, 
Hammond, A.
, 
Hendriks, M.
, 
Kgolo, T.
, 
Serrano, Y.
, 
Marquine, M. J.
, 
Dutt, A.
, 
Evans, J.
, & 
Judd, T.
 (2024). Neuropsychological application of the international test commission guidelines for translation and adapting of tests. Journal of the International Neuropsychological Society, 30(7), 621–634. 10.1017/S1355617724000286
39291438
PMC12565511

Noffs, M. H. S.
, 
Magila, M. C.
, 
Dos Santos, A. R.
, & 
Marques, C. M.
 (2002). Avaliação neuropsicológica de pessoas com epilepsia: Visão crítica dos testes empregados na população brasileira. Revista Neurociências, 10(2), 83–93.

Ono, S. E.
, 
de Carvalho Neto, A.
, 
Joaquim, M. J. M.
, 
Dos Santos, G. R.
, 
de Paola, L.
, & 
Silvado, C. E. S.
 (2019). Mesial temporal lobe epilepsy: Revisiting the relation of hippocampal volumetry with memory deficits. Epilepsy and Behavior: E&B, 100(A), 106516. 10.1016/j.yebeh.2019.106516
31574430

Ono, S. E.
, 
Mader‐Joaquim, M. J.
, 
de Carvalho Neto, A.
, 
de Paola, L.
, 
Dos Santos, G. R.
, & 
Silvado, C. E. S.
 (2021). Relationship between hippocampal subfields and verbal and visual memory function in mesial temporal lobe epilepsy patients. Epilepsy Research, 175, 106700. 10.1016/j.eplepsyres.2021.106700
34175793


Paštrnák, M.
, 
Sedláková, K.
, 
Dorazilová, A.
, & 
Rodriguez, M.
 (2018). Alternative forms parallel to the Czech versions of Rey Auditory Verbal Learning Test, complex figure test and verbal fluency. Česká A Slovenská Neurologie A Neurochirurgie, 81/114(1), 73–80. 10.14735/amcsnn201873


Putcha, D.
, 
Brickhouse, M.
, 
Wolk, D. A.
, 
Dickerson, B. C.
, & Alzheimer's Disease Neuroimaging Initiative
. (2019). Fractionating the Rey Auditory Verbal Learning Test: Distinct roles of large‐scale cortical networks in prodromal Alzheimer's disease. Neuropsychologia, 129, 83–92. 10.1016/j.neuropsychologia.2019.03.015
30930301
PMC6571167

Rasmussen, L. S.
, 
Larsen, K.
, 
Houx, P.
, 
Skovgaard, L. T.
, 
Hanning, C. D.
, 
Moller, J. T.
, & ISPOCD group. The International Study of Postoperative Cognitive Dysfunction
. (2001). The assessment of postoperative cognitive function. Acta Anaesthesiologica Scandinavica, 45(3), 275–289. 10.1034/j.1399-6576.2001.045003275.x
11207462


Rehnberg, J.
, 
Fors, S.
, 
Ford, K. J.
, & 
Leist, A. K.
 (2024). Cognitive performance trends among European older adults: Exploring variations across cohorts, gender, and educational levels (2007–2017). BMC Public Health, 24(1), 1646. 10.1186/s12889-024-19123-3
38902637
PMC11188163

Rey, A.
 (1958). L'examen clinique en psychologie. Presses Universitaires de France.

Salthouse, T. A.
 (2012). Robust cognitive change. Journal of the International Neuropsychological Society, 18(4), 749–756. 10.1017/S1355617712000380
22595019
PMC3633558

Salthouse, T. A.
 (2019). Trajectories of normal cognitive aging. Psychology and Aging, 34(1), 17–24. 10.1037/pag0000288
30211596
PMC6367038

Sanderson‐Cimino, M.
, 
Chen, R.
, 
Tu, X. M.
, 
Elman, J. A.
, 
Jak, A. J.
, & 
Kremen, W. S.
 (2023). Misinterpreting cognitive change over multiple timepoints: When practice effects meet age‐related decline. Neuropsychology, 37(5), 568–581. 10.1037/neu0000903
37079809
PMC10313772

Saury, J. M.
, & 
Emanuelson, I.
 (2017). Neuropsychological assessment of hippocampal integrity. Applied Neuropsychology. Adult, 24(2), 140–151. 10.1080/23279095.2015.1113536
27045585


Schwartzman, S.
 (2004). Equity, quality and relevance in higher education in Brazil. Anais da Academia Brasileira de Ciências, 76(1), 173–188. 10.1590/S0001-37652004000100015


Seblova, D.
, 
Berggren, R.
, & 
Lövdén, M.
 (2020). Education and age‐related decline in cognitive performance: Systematic review and meta‐analysis of longitudinal cohort studies. Ageing Research Reviews, 58, 101005. 10.1016/j.arr.2019.101005
31881366


Smirni, D.
, 
Smirni, P.
, 
Di Martino, G.
, 
Cipolotti, L.
, 
Oliveri, M.
, & 
Turriziani, P.
 (2018). Standardization and validation of a parallel form of the verbal and non‐verbal recognition memory test in an Italian population sample. Neurological Sciences, 39(8), 1391–1399. 10.1007/s10072-018-3433-z
29728938


Speer, P.
, 
Wersching, H.
, 
Bruchmann, S.
, 
Bracht, D.
, 
Stehling, C.
, 
Thielsch, M.
, 
Knecht, S.
, & 
Lohmann, H.
 (2014). Age‐ and gender‐adjusted normative data for the German version of Rey's auditory verbal learning test from healthy subjects aged between 50 and 70 years. Journal of Clinical and Experimental Neuropsychology, 36(1), 32–42. 10.1080/13803395.2013.863834
24341534


Witt, J. A.
, 
Coras, R.
, 
Becker, A. J.
, 
Elger, C. E.
, 
Blümcke, I.
, & 
Helmstaedter, C.
 (2019). When does conscious memory become dependent on the hippocampus? The role of memory load and the differential relevance of left hippocampal integrity for short‐ and long‐term aspects of verbal memory performance. Brain Structure and Function, 224(4), 1599–1607. 10.1007/s00429-019-01857-1
30863886


Zucchella, C.
, 
Federico, A.
, 
Martini, A.
, 
Tinazzi, M.
, 
Bartolo, M.
, & 
Tamburin, S.
 (2018). Neuropsychological testing. Practical Neurology, 18(3), 227–237. 10.1136/practneurol-2017-001743
29472384


## Supporting information


Table S1.

## Data Availability

The data sets generated and/or analysed during the current study are available from the corresponding author on reasonable request, in accordance with ethical guidelines.
