# Supplementary material for: Comparisons between two adapted versions of the Rey Auditory Verbal Learning Test in Brazilian adults: Effects of age and education
Source: J Neuropsychol. 2025 Nov 5;20(1):175–85. doi: 10.1111/jnp.70020 (PMC12976817; doi:10.1111/jnp.70020)
Supplement: Supplementary file 1 — Table S1. [file JNP-20-175-s001.docx]

**Supplementary Table S1.** Linear regression models for RAVLT-A variables A7 and SUM (A1-A5) adjusted for age, education level, and protocol.

|  | **R^2^** | **Classification** | **p*** | **Coefficients (95% CI) *** |
| --- | --- | --- | --- | --- |
| **SUM (A1-A5)** |  |  |  |  |
| Age (years) |  | ≥40 years(ref) |  |  |
|  |  | <40 years | <0.001 | 5.29 (3.29; 7.3) |
| Education level (years) |  | ≤ 9 (ref) |  |  |
|  |  | 10-12 | <0.001 | 6.33 (3.8; 8.85) |
|  |  | >12 | <0.001 | 11.98 (9.44; 14.52) |
| Protocol |  | B (ref) |  |  |
|  |  | A | 0.323 | 1.00 (-0.99; 3.00) |
| **A7** |  |  |  |  |
| Age (years) |  | ≥40 years(ref) |  |  |
|  |  | <40 years | <0.001 | 1.20 (0.60; 1.79) |
| Education level (years) |  | ≤ 9 (ref) |  |  |
|  |  | 10-12 | <0.001 | 1.76 (1.01; 2.50) |
|  |  | >12 | <0.001 | 3.21 (2.46; 3.96) |
| Protocol |  | B (ref) |  |  |
|  |  | A | 0.701 | 0.12 (-0.47; 0.70) |

Note: R^2^: Adjusted coefficient of determination; *Multivariate Linear Regression Model. p<0.05 (interactions between age and education level were not statistically significant); ref = reference; SUM (A1-A5) = Sum of the totals of the 5 trials (A1 to A5).
